# A Quantitative Comparison of Oil Sources on Shorelines of Prince William Sound, Alaska, 17 Years After the *Exxon Valdez* Oil Spill

**DOI:** 10.1007/s00244-023-01019-9

**Published:** 2023-08-16

**Authors:** Jeffrey W. Short, Jacek M. Maselko

**Affiliations:** 1 JWS Consulting LLC, 19315 Glacier Highway, Juneau, AK 99801 USA; 2 Auke Bay Laboratory, Alaska Fisheries Science Center, National Marine Fisheries Service, NOAA, 17315 Point Lena Loop Road, Juneau, AK 99801 USA

## Abstract

Environmental damage caused by accidental discharges of pollutants depends in part on the degree of prior contamination, in that increased pollution of an already heavily contaminated region will usually be considered less detrimental than equivalent pollution of a pristine region. Quantitative comparisons of specific pollution events with the extent and severity of prior contamination are rare, owing to difficulties in identifying and assessing contaminants remaining from prior pollution events, and in some cases contaminants from natural sources. The 1989 *Exxon Valdez* oil spill (EVOS) presents an unusual opportunity to quantitatively evaluate residual contaminants from petroleum sources on shorelines of Prince William Sound (PWS), Alaska. Here, we evaluate surface oil contamination from Monterey Formation petroleum-derived residues (released into PWS from ruptured storage tanks during the 1964 earthquake) on 200 shoreline segments selected at random within the EVOS spill path. We compare these results with previously estimated contamination from the EVOS and from other human activities. Our results indicate that residual shoreline contamination from the EVOS is more than ~ 50% greater than the sum total from human activity sites, that residual contamination by Monterey Formation sources is negligible in comparison to that from the EVOS, and that most of the shorelines in PWS were as close to pristine prior to the EVOS as is likely to be found anywhere else worldwide.

**Supplementary Information:**

The online version contains supplementary material available at 10.1007/s00244-023-01019-9.

Proper evaluation of environmental damage caused by accidental discharges of pollutants such as oil spills requires careful consideration of prior contamination. Pre-existing pollutant burdens provide context necessary for apportioning habitat degradation to the latest influx in comparison with ongoing degradation caused by previous contamination events. Because oil spills are so frequent and their residues may be persistent (NASEM 2022), resolving their contributions to the cumulative burden present in contaminated environments poses an enduring challenge. Even in remote regions, prior sources of oil may be contributing to ongoing habitat degradation.

Parties responsible for a particular spill have clear incentives to ensure that other sources are recognized to reduce their likelihood of being held responsible for contamination caused by others. An appropriate first step is therefore to identify all the sources. A logical next step is to quantify these prior pollution contributions. However, this second step is rarely taken because of the difficulties associated with making these quantitative estimates.

The 1989 *Exxon Valdez* oil spill (EVOS) provides an example that is amenable to a quantitative resolution. Although the accident released about 40,900 m^3^ of oil into Prince William Sound (PWS), contaminating shorelines with an estimated ~ 17,000 m^3^ initially (Wolfe et al. 1994), some of these shorelines had already been contaminated by previously released oil. Prior sources include (1) oil derived from the Monterey Formation in California that was released from storage tanks damaged by a magnitude 9 + earthquake in PWS in 1964 (Kvenvolden et al. 1995; Page et al. 2006; Wooley 2002); (2) oil released from other human activities such as mining, mineral prospecting, fish processing plants, mink and oyster farming, etc. (Wooley 2002); and (3) other undocumented marine oil spills. Oil residues from human activity (HA) sites have been estimated to have contaminated at least 3.6 ha, based on a comprehensive survey of shorelines in the area impacted by the EVOS (Page et al. 2006). Undocumented marine oil spills in the region involved light fuels such as diesel and gasoline from commercial fishing and recreational vessels that do not produce persistent residues (NASEM 2022). However, the volume of oil products released from storage tanks damaged by the 1964 earthquake was undoubtedly substantial based on the number and sizes of the tanks damaged in the Port of Valdez, Alaska (Kvenvolden et al. 1995). Kvenvolden et al. (1993) have reported that residues of this oil were easier to find on shorelines in Prince William Sound than residues from the EVOS, although Short et al. (2004) found that on surfaces of shorelines contaminated by the EVOS, surface oil from that spill was more prevalent than Monterey Formation oil.

Residual EVOS oil on shorelines has been estimated as 11.3 ha based on random sampling, with a 95% confidence interval of 6.78–17.2 ha (Short et al. 2004). Our objective here is to apply comparable methods to estimate the remaining amount of oil from the tanks damaged by the 1964 earthquake on these same shorelines, together with shorelines un-oiled by the EVOS. These results enable a quantitative comparison of the persistent residues from the three major sources (EVOS, HA and tanks damaged by the earthquake), providing context for evaluating the long-term impacts of the EVOS.

## Methods

### Study Area

**Fig. 1:**
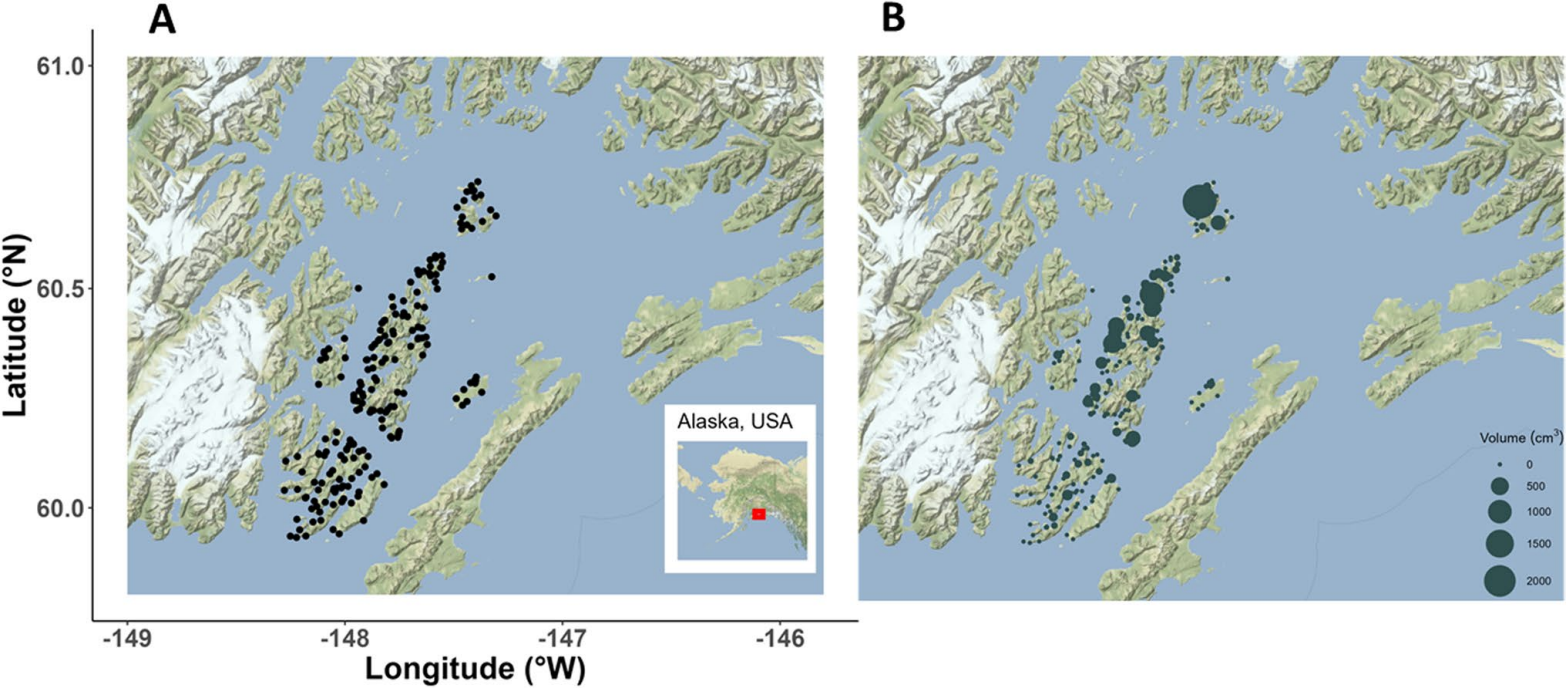
**A** Study area and region, showing locations of the 200 shoreline segments selected for visual evaluation of surface oil. **B** Total surface oil volumes (cm^3^) on beach segments within our study area

Our study area is defined by the islands of southwestern PWS within the trajectory of the EVOS, enclosed within the boundary shown in Fig. 1. This area encompassed 1,240,025 m of shoreline.

### Site Selection

The 1,240,025 m of shoreline within our study area was divided into segments of a maximum length of 100 m with several less than 100 m islets and leftover segments as identified using ArcView version 8.1 mapping software. Two hundred of these segments were chosen at random with selection probabilities proportional to the length of the segment, resulting in 19,687 m of shoreline selected for sampling.

### Oil Survey Methods

In 2006, 98 segments were sampled, mostly in the southern half of the sampling area, with the remaining 102 segments sampled during the summer of 2007. In the field, the beginning of each shoreline segment was identified using a hand-held Global Positioning System (GPS) receiver and accessed using a small inflatable boat. The shoreline segment length was measured using a 100-m survey tape along the high tide line. The surveys took place at low to mid-tide levels, based on recognition that Monterey Formation tar balls and tar mats have been most frequently found in the upper intertidal, well above the mid-tide level (Kvenvolden et al. 1995; Short et al. 2004).

Each shoreline segment was visually examined for the presence of surface oil from the water line to the vegetation line along the length of the segment by two independent observers, with each observer examining the whole segment area twice, from one end to the other and then back again. On 13 segments, the two observers kept separate notation of oil deposits for evaluation of differences in detection frequencies. We were unable to survey 7 segments on foot because they were nearly vertical rock walls, so we examined these from a small boat getting as close to the rock walls as possible.

**Fig. 2:**
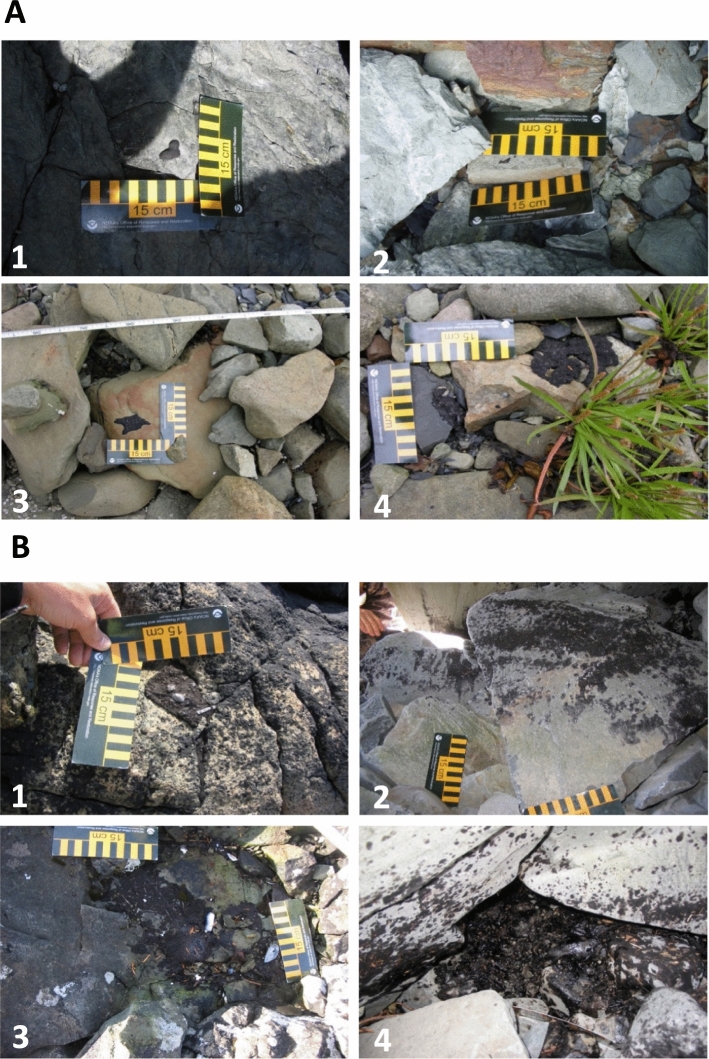
Typical deposits of tar balls and tar mats from **A** Monterey Formation oil tar ball deposits (1, 2, & 3) and tar mat deposits (4), and **B** Exxon Valdez oil tar ball (1), surface oil coats (2), tar mats (3), and asphalt pavements (4) on shorelines of Prince William Sound, Alaska

Monterey Formation tar balls and tar mats were primarily identified by visual characteristics. These tar balls typically present as small black splotches of highly-weathered oil on rocks, or less frequently as small (< 0.25 m^2^) tar mats, above the + 3 m tide height (Fig. 2A; Kvenvolden et al. 1995; Short et al. 2004). Surface oil deposits from the EVOS were distributed more widely throughout the upper intertidal zone and typically consisted of brown- to black-colored oil remaining as a thin coat or tar balls on rocks, as sometimes extensive (>> 0.25 m^2^) asphalt pavements, or as surface tar balls on rocks that were sometimes difficult to distinguish from Monterey Formation tar balls (Fig. 2B). During our surveys, we limited our search to tar balls, surface oil coats, tar mats and asphalt pavements less than 0.25 m^2^ in area of each contiguous occurrence. Within these limits, oil deposits that appeared to be EVOS oil were noted as such. In 14 questionable cases, we confirmed the source of the oil based on the ratio of di- and trialkyl-substituted dibenzothiophenes to, respectively, similar substituted phenanthrenes/anthracenes (i.e. C2-dibenzothiophenes:C2-phenanthrene/anthracenes, and C3-dibenzothiophenes:C3-phenanthrene/anthracenes, where “C2” and “C3” indicate the number of carbon atoms in the alkyl substituents), determined by gas chromatography-mass selective detector (GCMS) analysis of the oils as described in Short et al. (1996). The GCMS analysis method in Short et al. (1996) is nearly identical with that used by Page et al. (2006) (See Online Resource 1 for details). The ratios of C2-dibenzothiophenes:C2-phenanthrene/anthracenes, and of C3-dibenzothiophenes:C3-phenanthrene/anthracenes, for EVOS oil is ~ 0.8 to  ~ 1.0 (Bence et al. 1996; Short et al. 1996), whereas the comparable ratios for two samples of Monterey Formation oil ranged from 0.24 to 0.30 (Bence et al. 1996). This same analytical method (Short et al. 1996) was used by Short et al. (2004) to evaluate source oils in their study that estimated the extent of residual oil contamination from the EVOS on PWS shorelines. See Online Resource 1 for additional details comparing the methods used in this study, Short et al. 2004, and Page et al. 2006.

### Measurement of Oil on Shorelines

Surface oil was documented by measuring the approximate mean thickness of oil patches with a ruler and by photographing each patch from at least two separate angles with a ruler in view to calculate the surface area (Fig. 2). Able Image Analyser (Mu Labs; mulabs.com) was used to digitize each photo and measure the surface area of each oil patch. Volume was calculated as the product of the area and the estimated mean thickness of each oil patch. See Online Resource 2 for the results of these measurements, along with distance to and oiling intensities at the nearest EVOS shoreline segment evaluated during the shoreline cleanup assessment technique surveys conducted in the immediate aftermath of the EVOS, the distance to the nearest HA site, and site characteristics for each of the 200 beach segments we surveyed. The relation between tarball area and volume did not appear to differ with proximity to oiled EVOS shoreline segments (Fig. S1, Online Resource 2). The relation of the 200 shoreline segments evaluated for this study to the HA sites evaluated by Page et al. (2006) is depicted in Fig. S2, Online Resource 2.

### Statistical Estimation of Oil Volume and Mass

The total surface area of oil *T* in the sampling region of Prince William Sound was calculated using the Hansen–Hurwitz estimator (Thompson, 1992):

$$\hat{T} = \frac{L}{n}\sum\limits_{i = 1}^{n} {\frac{{y_{i} }}{{L_{i} }}}$$

where *L* is the total shoreline length, *n* is the number of segments sampled (= 200), *L*_*i*_ is the length of the *i*th shoreline segment and *y*_*i*_ is the total oil area found on the *i*th shoreline segment. Variance was computed as:

$$\hat{V}(\hat{T}) = \frac{{L^{2} }}{n(n - 1)}\sum\limits_{i = 1}^{n} {\left( {\frac{{y_{i} }}{{L_{i} }} - \frac{{\hat{T}}}{L}} \right)}^{2}$$

Oil volume was calculated in the same way, except *y*_*i*_ was replaced by the oil volume found on the *i*th shoreline segment, obtained by summing across all oil patches (*M*_*i*_) found on segment *i*, the product of each oil patch area (*a*_*ik*_) and average thickness of that oil patch (*d*_*ik*_):

$$y_{i} = \sum\limits_{k = 1}^{{M_{i} }} {a_{ik} d_{ik} }$$

## Results

We found oil in 74 of the 200 segments sampled. Oil patches (e.g. Figure 2) on a single segment ranged from 0.42 cm^2^ containing 0.084 cm^3^ of oil to 2902 cm^2^ containing 2369 cm^3^ of oil. Local tar ball and tar mat deposits ranged from 0.29 cm^2^ containing 0.029 cm^3^ of oil to 832 cm^2^ containing 576 cm^3^ of oil and 302 cm^2^ containing 905 cm^3^ of oil. The total area of oil observed was 1.323 m^2^ and the total volume was 0.00837 m^3^. The total estimated area of oil in our study area was 96.9 m^2^ (95% confidence interval: 35.6–158.1 m^2^), and the total volume was 0.639 m^3^ (95% confidence interval: 0.153–1.124 m^3^).

A total of 51 tar ball or tar ball clusters were detected on the 13 segments where observers kept separate notation of oil deposits for evaluation of differences in detection frequencies. Twenty-seven of these were detected by both observers, 13 by observer #1 but not by observer #2, 9 by observer 2 but not by observer 1, and 2 that were not detected by either observer until after they had shared their collection notes and revisited areas where one of the observers failed to detect tar balls found by the other. Thus, the empirical detection probability of observer #1 is 40/51 = 0.784 while that of observer #2 is 36/51 = 0.706, implying that the probability of both observers failing to detect tar balls is (1–0.784) (1–0.706) = 0.0635. This broadly agrees with the 2 of 51 = 0.039 frequency of tar ball or tar ball clusters that both actually failed to observe.

Monterey Formation tar balls and tar mats accounted for 88.5% of the total oiled area and 90.4% of the total oiled volume. Thirteen of the 14 tar balls where the oil source was not clear based on visual appearance were confirmed as Monterey Formation in origin, with C2-dibenzothiophenes:C2-phenanthrene/anthracenes and of C3-dibenzothiophenes:C3-phenanthrene/anthracenes ratios that ranged from 0.129 to 0.387 and from 0.114 to 0.452, respectively. The remaining tar ball was EVOS, with the ratio of C3-dibenzothiophenes:C3-phenanthrene/anthracenes of 1.17. This sample was so weathered that the concentrations of C2-dibenzothiophenes and of C2-phenanthrene/anthracenes were below method detection limits.

## Discussion

Our results show that the area covered by Monterey Formation tar balls and tar mats scattered throughout shorelines that are removed from current or historical HA sites are a small proportion of the oil-contaminated area of HA sites, and an even smaller proportion of shorelines contaminated by the EVOS (Table 1). Of the 96.9 m^2^ of tar balls and tar mats that we estimated on shorelines in this study, approximately 88.5% were Monterey Formation oil, implying a shoreline contamination area from this oil source of (96.9 m^2^) (0.885) = 85.8 m^2^, or 0.00858 ha. The area of contaminated HA shorelines as estimated by Page et al. (2006) of 3.6 ha is greater by a factor of 420, and the 11.3 ha area of EVOS-contaminated shorelines as estimated by Short et al. (2004) is greater by a factor of 1300.

**Table 1:** Estimated surface-oiled areas and amounts on shorelines of Prince William Sound, Alaska, from the 1989 Exxon Valdez oil spill (from Short et al. 2004), from human activity sites prior to and within the trajectory of the Exxon Valdez oil spill (from Page et al. 2006), and from Monterey Formation oil discharged into Prince William Sound from oil product storage tanks ruptured by the 1964 Alaska earthquake (this study)

	Exxon valdez oil	Human activity sites	Monterey formation oil
Oiled areas (ha)	11.31	3.6	0.0086
Oil mass (kg)	83,400	(0.46)*	639

Data supporting direct comparison of oil masses from the Exxon Valdez oil spill with the human activity sites are not available

*Estimated ratio of TPAH at Human Activity sites and Exxon Valdez oil

Comparison of oil contamination in terms of amounts are complicated by the fact that oil volumes at HA sites were not estimated in the Page et al. (2006) study. Short et al. (2004) estimated that 55,600 kg of subsurface EVOS oil remained in the mid- to upper intertidal in 2001, and a subsequent study implies that half again as much subsurface oil was present in the lower intertidal (Short et al. 2006), for a total of (55,600 kg) (1.5) = 83,400 kg. This is approximately 130 times larger than the amount of Monterey Formation oil estimated in this study (0.639 m^3^ oil $\cong$ 639 kg oil, assuming an oil density of 1000 kg/m^3^; Table 1). While amounts of oil from the EVOS cannot be directly compared with amounts of oil at HA sites, a comparison in terms of total polycyclic aromatic hydrocarbons (TPAH) can be made. Page et al. (2006) report that 36,000 m^2^ of contaminated shoreline sediments were sampled to depths of 0.25–0.50 m and analyzed for TPAH by GC–MS. The average TPAH concentration of these sediments, weighted by the contaminated area at each sampled location, is 19.7 mg TPAH/kg sediment. Assuming a sediment density of 1,600 kg/m^3^, the mass of sediment sampled during the Page et al. (2006) study is (36,000 m^2^) (0.50 m)(1600 kg/m^3^) = 2.88 × 10^7^ kg, containing (2.88 × 10^7^ kg)(19.7 mg TPAH/kg) = 5.67 × 10^8^ mg TPAH, or 567 kg TPAH. In comparison, the TPAH concentration of weathered EVOS oil is about 1.48% (Wang et al. 2003), implying at TPAH mass of 1230 kg (= 83,400 kg EVOS oil) (0.0148 kg TPAH/kg EVOS). This is more than twice the estimated 567 kg TPAH at HA sites, and does not include additional TPAH from surface EVOS oil.

The 567 kg TPAH estimated at the combined total of the nine HA sites surveyed by Page et al. (2006) is clearly an underestimate, as these authors assert that there are more than 50 HA sites in the sound, but the magnitude of this underestimation is unclear. Page et al. (2006) state that the nine locations they surveyed represent the range of historical activities in the sound, and suggest that “Because there are many more HA sites in PWS than those surveyed, it is likely that the total area of contaminated sediment is substantially more than the 8.9 acres [i.e. 36,000 m^2^] found at the nine sites surveyed.” While the nine sites surveyed by Page et al. (2006) include mines, an ore processing facility, fish processing facilities and canneries, and a former settlement, the sites selected are not necessarily representative of their respective categories. The two surveyed mine sites were by far the largest and most commercially-successful mines in the sound, accounting for 20,500 m^2^ of the 36,000 m^2^ of contaminated shoreline of all nine sites combined. Of all the mine sites along the EVOS spill path in western PWS identified in Fig. 1 of Page et al. (1999) but not surveyed by Page et al. 2006, none were commercially successful (Lethcoe and Lethcoe 2001). Only one included an ore processing facility, consisting of a crude Chilean Mill that produced 1.93 kg of gold and 1.62 kg of silver (minedat.org). More generally, mines here and elsewhere in PWS that were not included among the nine sites surveyed by Page et al. (2006) were almost always located on uplands removed from shorelines, so that their cumulative contribution to PAH contamination of shorelines in PWS is likely negligible. Nearly all of the historically-operated fish processing/cannery plants identified in Fig. 1 of Page et al. (1999) were included in the Page et al. (2006) study. The Alaska Native village of Chenega, one of the former settlements identified by Page et al. (1999), was destroyed by a tidal wave generated by the 1964 Alaska earthquake, which likely also dispersed any PAH-contaminated shoreline sediments there. Other former settlements in the region apart from those at the Latouche mine and along the adjacent Sawmill Bay on Elrington Island were small and ephemeral, usually consisting of 20 or fewer inhabitants (Lethcoe and Lethcoe 2001). One small artisinal sawmill in Thumb Bay produced lumber for uses apart from building materials (Lethcoe and Lethcoe 2001). Finally, the fish hatcheries in the region were built in the mid-1970’s and are subject to modern fuel management regulations. Considering all this, we believe that the total impact of all prior HA sites within the trajectory of the EVOS was not more than about 25% greater than the estimates based on the nine sites surveyed by Page et al. (2006), or contaminated shoreline area of about (1.25) (36,000 m^2^) = 45,000 m^2^ and a combined TPAH mass of (1.25) (567 kg TPAH) = 709 kg TPAH.

Our results show that the EVOS is likely the largest source of current long-term hydrocarbon contamination on PWS shorelines within the EVOS spill path, exceeding the combined total of HA sites by a substantial margin and far exceeding Monterey Formation tar balls and tar mats. On the islands within our study area in PWS, the shorelines contaminated by the EVOS were mostly pristine with respect to hydrocarbon pollution prior to the 1989 spill. With a maximum tidal excursion of 5 m and assuming an average shoreline slope of 45º, the 1,240,025 m shoreline length in our study area would have an intertidal area of 8.77 × 10^6^ m^2^, of which the 21,550 m^2^ contaminated area of HA shorelines on these islands (Page et al. 2006) is about 0.25%. While shorelines along the northern Gulf of Alaska were historically affected by widespread but low-intensity human activities (Wooley 2002), shorelines of the islands that we surveyed were little affected by other human activities besides those at the HA sites included in the Page et al. (2006) study. Moreover, the magnitude 9 + 1964 Alaska earthquake raised 1–3 m of shoreline on these islands above the intertidal zone (Plafker 1969), which was replaced by pristine former subtidal seafloor in the lower intertidal. Analysis of pre-industrial aquatic sediment TPAH loadings measured by GCMS in sediment cores of lakes imply a planetary natural background on the order of several tens up to ~ 100 ng/g, composed primarily of PAH produced by combustion during forest and brush fires (Wakeham et al. 1980; Pereira et al. 1999; Ricking and Schulz 2002, Yunker et al. 2003, and references therein). Analysis of sediment samples collected from the 0 m tide level (i.e. mean lower low water) at reference sites in PWS sampled from 1989 to 1991 were usually near or below this loading threshold (O’Clair et al. 1996). We conclude that prior to the EVOS, most of the shorelines contaminated by the EVOS in PWS were as close to pristine as is likely to be found anywhere else worldwide.

## Supplementary Information

Below is the link to the electronic supplementary material.

Supplementary file1 (DOCX 23 KB)

Supplementary file2 (XLSX 1881 KB)
