# Supplementary material for: A Quantitative Comparison of Oil Sources on Shorelines of Prince William Sound, Alaska, 17 Years After the Exxon Valdez Oil Spill
Source: Arch Environ Contam Toxicol. 2023 Aug 16;85(2):140–6. doi: 10.1007/s00244-023-01019-9 (PMC10447268; doi:10.1007/s00244-023-01019-9)
Supplement: Supplementary file 1 — Supplementary file1 (DOCX 23 KB) [file 244_2023_1019_MOESM1_ESM.docx]

ONLINE RESOURCE 1

Comparison of Methods Used by Three Recent Studies to Quantitatively Evaluate Long-Term Hydrocarbon Contamination of Shorelines in Prince William Sound, Alaska

Supplementary Information

for

A Quantitative Comparison of Oil Sources on Shorelines of Prince William Sound, Alaska, 17 Years after the *Exxon Valdez* Oil Spill

Published in

Archives of Environmental Contamination and Toxicology

by

JEFFREY W. SHORT*^1^, JACEK M. MASELKO^2^

^1^JWS Consulting LLC, 19315 Glacier Highway, Juneau, Alaska 99801

^2^Auke Bay Laboratory, Alaska Fisheries Science Center, National Marine Fisheries Service, NOAA, 17315 Point Lena Loop Road, Juneau, Alaska 99801

*Corresponding author telephone: 907-209.3321; fax: 907-586-4944; e-mail: jwsosc@gmail.com

I.

Table S1. Comparison of methods used by two studies along with this study to evaluate lingering oil or PAH/PAC contamination on shorelines of Prince William Sound after the 1989 Exxon Valdez oil spill.

| **Study** | | | |
| --- | --- | --- | --- |
| **Study Descriptor** | Short et al. 2004 | Page et al. 2006 | This Study |
| Study  Design | Two-level stratified random sampling (SRS) from mid- through upper intertidal within each of three 1989 – 1992 shoreline oiling intensity categories. Level I: 91 total shoreline segments, randomly selected within each oiling intensity category; Level II: randomized placement of 0.25 m^2^ sample quadrats within each shoreline. 7,484 quadrats sampled, excavated to 0.5 m depth when possible | Delineation of total PAH contamination area at 9 of the most contaminated historical human activity sites within or near the EVOS spill path. Delineation based on colorimetric screening results of solvent-extractible organic matter from sediment samples collected along the vertical wall of test pits excavated to 0.5 m depth when possible at intersections of a grid network with 10 m x 10 m cells covering the area of positive screening test results. Additional pits were excavated at closer spacings within the contaminated area of the grid | 200 shoreline segments each 100 m or less in length randomly selected from 1,240 km of shorelines of islands along EVOS spill path. Lower through upper intertidal inspected twice by each of two observers for surface tarball occurrences only |
| Precision Estimate | Yes | No | Yes |
| Initial Oil Detection Method | Visual or olfactory | Visual | Visual |
| Oil Quantification Methods | Oiled area statistically extrapolated from SRS results; oil mass based oiling intensity category of each pit and the average oil mass extracted and gravimetrically determined in all the sediments excavated within each 0.25 m^2^ quadrat for each of the three shoreline oiling intensity categories. Total gravimetric determinations = 97. | Oiled area estimated by Kriging interpolation of colorimetric test results to a limit of 2.5 ppm TPAH. Sediment loadings of TPAH were determined immunochemically on a wet sediment weight basis, and by GCMS on a dry sediment weight basis. | Tarball and mat areas estimated by digitizing image analyzer of photographs with distance ruler included. Average thickness estimated using a hand ruler, and oil volume estimate as the product of oiled area and thickness. Oil mass assumed an oil density of 1 g/mL |
| Oil and PAH/PAC Fingerprinting | Oil source determination based on petroleum biomarker presence and ratios of typical occurrences of 27 surface and 38 subsurface oil deposits. Exxon Valdez oil was identified on the basis of the joint absence of  17α(H),18α(H),21β(H)-28,30-bisnorhopane and of 18(α+β)-  (H)-oleanane, and ratios near 2 of Triplet (C_26_-tricyclic terpane  (S?)+C_26_-tricyclic terpane (R?))/C_24_-tetracyclic terpane measured by GCMS at m/z 191. Monterey Formation contained bisnorhopane  and oleanane and had ratios near 4 of Triplet. | PAH source identification was based on relative concentrations of PAH and PAC detected by GCMS.  The GCMS analysis include 35 2- through 6-ring parent PAH C1 through C3 or C4 alkyl homologues, the parent and C1 through C3 alkyl homologues of dibenzothiophene, and dibenzofuran (listed in Table 1, Page et al. 1995) | PAH source identification was based on relative concentrations of PAH and PAC detected by GCMS. Excepting dibenzofuran, the PAH and PAC analytes were the same as those used by Page et al. (1995). Discrimination between Exxon Valdez and Monterey Formation oil sources based on ratios of C2-dibenzothiophenes:C2-phenanthrene/  anthracenes and C3-dibenzothiophenes:C3-phenanthrene/  anthracenes |
| Study Limitations | Only sampled sediments within +1.8 m – +4.8 m tidal elevations above mean lower low water | Only sampled 9 of >50 historical human activity sites in PWS, method used for TPAH source apportionment not clear, and did not estimate mass of TPAH contamination | Did not evaluate sites that may have been contaminated by Monterey Formation tarballs and tar mats on mainland shorelines |

Abbreviations: GCMS - gas chromatography-mass selective detection, EVOS - Exxon Valdez oil spill, PAH - polycyclic aromatic hydrocarbon, PAC - polycyclic aromatic compound, PWS - Prince William Sound, TPAH - total polycyclic aromatic hydrocarbons and compounds combined.

II. Comparison of methods used to identify oil sources following the 1989 Exxon Valdez oil spill

Bence et al. (1996) compared the advantages and disadvantages of the methods that have been used to identfy sources of whole oil, aliphatic or aromatic hydrocarbons following the Exxon Valdez oil spill (EVOS) in their Table 3. Of these, three had both high environmental stability (i.e. were little affected by changes in oil composition caused by weathering processes), and high source specificity. These three methods are: (1) carbon isotopic composition (δ^13^C) measured by isotope ratio mass spectrometry; (2) aliphatic saturate petroleum biomarkers measured by GCMS; and (3) measurement of triaromatic stearanes by GCMS. A fourth method, analysis of PAH/PAC, has variable environmental stability and moderate to high specificity. In their Fig. 6, Bence et al. (1996) showed that ratios of C2-dibenzothiophenes:C2-phenanthrene/anthracenes and of C3-dibenzothiophenes:C3-phenanthrene/anthracenes clearly distinguished Exxon Valdez oil from Monterey Formation oil sources. Subsequently, Douglas et al. (1996) showed that these ratios distinguished PAH/PAC from these two sources even after weathering losses of 98% of total PAH. Recognizing that the tarballs encountered in our study here almost certainly were from one or the other of these two sources, we used these ratios to distinguish between them as a matter of analytical convenience, as PAH were routinely measured in environmental samples from the EVOS in our laboratory.

References

Bence AE, Kvenvolden KA, Kennicutt II MC (1996) Organic geochemistry applied to environmental assessments of Prince William Sound, Alaska, after the Exxon Valdez oil spill—a review. Org geochem 24:7–42.

Douglas GS, Bence AE, Prince RC, McMillen SJ, Butler EL (1996) Environmental stability of selected petroleum hydrocarbon source and weathering ratios. Environ Sci Technol 30:2332–2339.

Page DS, Boehm PD, Douglas GS, Bence EA (1995) Identification of hydrocarbon sources in the benthic sediments of Prince William Sound and the Gulf of Alaska following the Exxon Valdez oil spill. ASTM Special Technical Publication 1219:41-83.

Page DS, Brown JS, Boehm PD, Bence AE, Neff JM (2006) A hierarchical approach measures the aerial extent and concentration levels of PAH-contaminated shoreline sediments at historic industrial sites in Prince William Sound, Alaska. Mar Pollut Bull 52:367–379.

Short JW, Lindeberg MR, Harris PM, Maselko JM, Pella JJ, Rice SD (2004) Estimate of oil persisting on the beaches of Prince William Sound 12 years after the Exxon Valdez oil spill. Environ Sci Technol 38:19–25.
